# Risk factors associated with safety of preschool peanut oral immunotherapy

**DOI:** 10.1016/j.jacig.2023.100094

**Published:** 2023-03-21

**Authors:** Duva Karunakaran, Edmond S. Chan, Qian Zhang, Jeffrey N. Bone, Stuart Carr, Sandeep Kapur, Gregory A. Rex, Mary McHenry, Scott B. Cameron, Victoria E. Cook, Sara Leo, Tiffany Wong, Thomas V. Gerstner, Joanne Yeung, Elissa M. Abrams, Raymond Mak, Stephanie C. Erdle, Lianne Soller

**Affiliations:** a British Columbia Children’s Hospital, Vancouver, British Columbia; b Department of Medicine, Queen’s University, Kingston, Ontario; c Department of Pediatrics, Division of Allergy, University of British Columbia, Vancouver, British Columbia; d Snö Asthma & Allergy Clinic, Abu Dhabi, United Arab Emirates; e Department of Pediatrics, Division of Allergy, Dalhousie University/IWK Health Centre, Halifax; f Halifax Allergy & Asthma Associates, Halifax, Nova Scotia; g Community Allergy Clinic, Victoria, British Columbia; h West Coast Allergy and Immunology Clinic, Vancouver, British Columbia; i Department of Pediatrics, Section of Allergy and Clinical Immunology, University of Manitoba, Winnipeg, Manitoba; j Meadowood Medical Center, Winnipeg, Manitoba; k Vancouver Kids Allergy, Vancouver, British Columbia

**Keywords:** Oral immunotherapy, personalized medicine, shared decision making, peanut allergy

## Abstract

**Background:**

An understanding of how patient characteristics such as age, baseline peanut-specific IgE, and atopic comorbidities may influence potential safety outcomes during peanut oral immunotherapy (P-OIT) could aid in shared decision making between clinicians and patient families.

**Objective:**

This study explored the relationship between baseline patient characteristics and reactions during P-OIT using a large sample size to better understand potential risk factors influencing P-OIT safety.

**Methods:**

Data were obtained from the Food Allergy Immunotherapy (FAIT) registry, which collects real-world OIT data from community and academic allergy clinics across Canada. Multivariable logistic regression modeling was performed to examine the relationship between baseline patient characteristics and reactions during P-OIT. Multiple imputation was applied to reduce potential bias caused by missingness and to maximize the use of available information to preserve statistical power.

**Results:**

Between April 2017 and June 2021, a total of 653 eligible patients initiated P-OIT. Multivariable regression analysis showed pre-OIT grade 2+ initial reaction (odds ratio [OR] = 1.33, 95% confidence interval [CI] 1.10, 1.61), allergic rhinitis (OR = 1.60, 95% CI 1.08, 2.38), older age (OR = 1.01, 95% CI 1.00, 1.02), and higher baseline peanut-specific IgE (OR = 1.02, 95% CI 1.02, 1.03) were associated with grade 2+ reaction during P-OIT after adjusting for potential risk factors.

**Conclusion:**

Our study identified several clinically important risk factors for grade 2+ reactions during P-OIT: pre-OIT grade 2+ initial reaction, allergic rhinitis, older age, and higher baseline peanut-specific IgE. These results highlight the need for individualized risk stratification for OIT.

## Introduction

Growing evidence suggests that real-world preschool peanut oral immunotherapy (P-OIT) is safe and effective.^1^^,^^2^ However, anaphylaxis during P-OIT may occur. An understanding of how patient characteristics such as age, baseline peanut-specific IgE (sIgE), and atopic comorbidities may influence the potential safety outcomes during P-OIT could aid in shared decision making between clinicians and patient families.^3^

We explored the relationship between baseline patient characteristics and reactions during preschool P-OIT using a large sample size to better understand potential risk factors influencing P-OIT safety.

Data were obtained from the Food Allergy Immunotherapy (FAIT) registry, which collects real-world OIT data from community and academic allergy clinics across Canada.^1^^,^^2^ This study received ethics approval from the University of British Columbia (approval H20-02342).

Patients began receiving 1 to 12 mg of peanut protein depending on age, clinical history, and P-OIT protocol (ie, Bamba peanut snack only, capsules only, or hybrid), with dose escalations every 2 to 4 weeks until a maintenance dose of 300 mg protein was reached.^2^ Once at the maintenance phase, patients continued this dose for approximately 1 year, after which an exit oral food challenge targeting 4 g of peanut protein was conducted.^1^

Inclusion criteria included preschool patients (9-70 months) who completed P-OIT buildup or dropped out, with a history of objective reaction to peanut and positive skin prick test (SPT) wheal of ≥3 mm or peanut-specific IgE of ≥0.35 kU/L; if no history of peanut ingestion was present, then peanut-specific IgE was ≥5 kU/L.^2^ Exclusion criteria for this analysis included patients aged >70 months, patients who initiated >12 mg of peanut protein, and patients who were still updosing at the time of analysis.

For statistical analysis, univariate regression modeling and multivariable logistic regression modeling were performed to examine the associations of potential risk factors with the outcome of maximum grade of reaction (grade 2+ vs grade 1/no reaction) over the course of P-OIT (first-day escalation, P-OIT buildup, and P-OIT maintenance). This distinction was made because grade 2+ generally defines reactions that would affect the treatment plan.^4^

Potential risk factors in the regression models included the following binary variables: sex, pre-OIT grade 2+ initial reaction (vs grade 1 initial reaction or no history of reaction), starting dose 12 mg peanut protein (vs <12 mg), and presence of eczema, asthma, or allergic rhinitis at enrollment (Table I); and the following continuous variables: age at entry into P-OIT, baseline SPT, baseline sIgE, and number of total weeks of P-OIT from enrollment to date of last visit where safety could be assessed.

**Table I:** Baseline patient characteristics according to grade of reaction during P-OIT

Characteristic	Reaction grade		
	0-1	2+	Total
No. of subjects	429	224	653
Male sex	266 (62.0)	136 (60.7)	402 (61.6)
Pre-OIT grade 2+ initial reaction	107 (24.9)	93 (41.5)	200 (30.6)
Starting dose			
<12 mg	310 (72.3)	147 (65.6)	457 (70.0)
12 mg	119 (27.7)	77 (34.4)	196 (30.0)
At least 1 atopic condition	303 (70.6)	175 (78.1)	478 (73.2)
Eczema	283 (66.0)	157 (70.1)	440 (67.4)
Asthma	48 (11.2)	41 (18.3)	89 (13.6)
Allergic rhinitis	14 (3.3)	19 (8.5)	33 (5.1)
Age at start of P-OIT (months)	17.0 (11.0-27.0)	23.0 (12.0-39.0)	18.0 (11.0-31.0)
Baseline SPT (mm)	7.0 (5.0-9.0)	8.0 (6.0-10.0)	7.0 (5.0-10.0)
Baseline sIgE (kU/L)	3.0 (0.9-10.1)	14.2 (3.4-52.6)	4.9 (1.3-20.3)
Total no. of weeks of P-OIT from baseline to last visit date	15.2 (8.0-20.1)	14.3 (5.5-20.1)	14.8 (7.3-20.1)

Data are presented as nos. (%) or medians (interquartile ranges).

The effect estimates were reported as unadjusted and adjusted odds ratios (ORs) with 95% confidence intervals (CIs). All analyses were conducted with SAS 9.4 software (SAS Institute).

## Results and discussion

Between April 2017 and June 2021, a total of 653 eligible patients initiated P-OIT. Median (interquartile range) age at OIT entry was 18 (11.0-31.0) months (Table I). One third of patients had grade 2 reactions, no patients had grade 3 reactons, and 1.1% had grade 4 reactions during P-OIT.

The univariate model showed pre-OIT grade 2+ initial reaction, asthma, allergic rhinitis, age, baseline SPT, and baseline sIgE were associated with grade 2+ reaction during P-OIT (Table II).

**Table II:** Unadjusted and adjusted model with multiple imputations for relationship between grade 2+ reactions during P-OIT and risk factors

Variable	OR (95% CI) for:	
	Unadjusted model	AMMI model
No. of subjects	653	653
Sex (M/F)	0.95 (0.68, 1.32)	0.98 (0.81, 1.17)
Pre-OIT grade 2+ initial reaction	**2.14 (1.51, 3.01)**	**1.33 (1.10, 1.61)**
Starting dose of 12 mg (yes/no)	1.36 (0.96, 1.93)	1.15 (0.95, 1.40)
Eczema (yes/no)	1.21 (0.85, 1.71)	1.00 (0.82, 1.22)
Asthma (yes/no)	**1.78 (1.13, 2.80)**	0.92 (0.70, 1.21)
Allergic rhinitis (yes/no)	**2.75 (1.35, 5.59)**	**1.60 (1.08, 2.38)**
Age at start of P-OIT (months)	**1.02 (1.01, 1.03)**	**1.01 (1.00, 1.02)**∗
Baseline SPT	**1.09 (1.04, 1.14)** [n = 639]	1.03 (0.97, 1.08)
Baseline sIgE	**1.03 (1.02, 1.03)** [n = 467]	**1.02 (1.02, 1.03)**∗
Total no. of weeks of P-OIT from baseline to last visit date	0.99 (0.97, 1.01)	1.00 (0.98, 1.02)

∗ When applied to a clinical setting, an OR of 1.01 means a 1% higher risk of having a grade 2+ reaction during OIT per 1-month increase in age at initiation OIT. An OR of 1.02 means a 2% increase in risk of having a grade 2+ reaction during OIT per 1 kU/L increase in baseline sIgE. Bold indicates a statistically significant relationship between the outcome and risk factor (ie, the 95% CI does not cross null).

When comparing outcome and risk-factor variables between patients with and without missing data, differences could be observed among several variables, particularly pre-OIT grade 2+ initial reaction, starting dose, atopic conditions, and baseline sIgE (Table III). Because this may imply potential bias of estimates caused by exclusion of patients with missing values, we decided to account for missing data using multiple imputation via the adjusted model, multiple imputation (AMMI) model.

**Table III:** Comparison of independent and dependent variables between patients with versus without missing data

Variable	Missing data	
	No	Yes
No. of subjects	453	200
Grade 2+ reactions during P-OIT		
No	297 (65.6)	132 (66.0)
Yes	156 (34.4)	68 (34.0)
Sex		
Female	177 (39.1)	74 (37.0)
Male	276 (60.9)	126 (63.0)
Pre-OIT grade 2+ initial reaction		
No	303 (66.9)	150 (75.0)
Yes	150 (33.1)	50 (25.0)
Starting dose		
<12 mg	278 (61.4)	179 (89.5)
12 mg	175 (38.6)	21 (10.5)
Eczema		
No	130 (28.7)	83 (41.5)
Yes	323 (71.3)	117 (58.5)
Asthma		
No	380 (83.9)	184 (92.0)
Yes	73 (16.1)	16 (8.0)
Allergic rhinitis		
No	434 (95.8)	186 (93.0)
Yes	19 (4.2)	14 (7.0)
Age at start of P-OIT (months)	19.0 (11.0-34.0)	17.0 (12.0-27.0)
Baseline SPT (mm)	7.5 (6.0-10.0)	6.0 (5.0-8.0)
Baseline sIgE (kU/L)	5.0 (1.3-19.9)	4.8 (3.5-32.4)
Total no. of weeks of P-OIT from baseline to last visit date	14.5 (7.4-19.7)	16.0 (7.0-21.5)

Data are presented as nos. (%) or medians (interquartile ranges).

The AMMI model showed pre-OIT grade 2+ initial reaction (OR = 1.33, 95% CI 1.10, 1.61), allergic rhinitis (OR = 1.60, 95% CI 1.08, 2.38), older age (OR = 1.01, 95% CI 1.00, 1.02), and higher baseline sIgE (OR = 1.02, 95% CI 1.02, 1.03) were associated with grade 2+ reactions during P-OIT (Table II), after adjusting for potential risk factors.

Although there was a positive effect of allergic rhinitis on the outcome in the AMMI model as well as sensitivity analysis 2 (SA2) models, this was not observed in SA1 models (Table IV). There was no relationship between baseline SPT and outcome in the AMMI or SA1 models, but there was a positive relationship in the SA2 model. The effect estimates of other risk factors remained consistent between the main analysis and sensitivity analyses.

**Table IV:** Models for relationship between grade 2+ reactions and predictor variables

Variable	OR (95% CI) for:	
	Adjusted model without imputation (SA1)	Adjusted model with reduced variables (SA2)
No. of subjects	453	639
Sex (M/F)	0.91 (0.59, 1.42)	0.91 (0.64, 1.29)
Pre-OIT grade 2+ initial reaction	**1.74 (1.11, 2.72)**	**1.98 (1.38, 2.85)**
Starting dose of 12 mg (yes/no)	1.34 (0.86, 2.10)	1.24 (0.85, 1.80)
Eczema (yes/no)	0.91 (0.57, 1.47)	1.09 (0.75, 1.58)
Asthma (yes/no)	0.94 (0.51, 1.76)	1.15 (0.69, 1.92)
Allergic rhinitis (yes/no)	1.31 (0.47, 3.64)	**2.22 (1.01, 4.87)**
Age at start of P-OIT (months)	**1.01 (1.00, 1.03)**	**1.01 (1.00, 1.02)**
Baseline SPT (mm)	1.04 (0.98, 1.11)	**1.07 (1.02, 1.12)**
Baseline sIgE (kU/L)	**1.02 (1.01, 1.03)**	Not included
Total no. of weeks of P-OIT from baseline to last visit date	0.99 (0.97, 1.02)	0.99 (0.98, 1.01)

Bold indicates a statistically significant relationship between the outcome and risk factor (ie, the 95%CI does not cross null).

Our data suggest that pre-OIT grade 2+ initial reaction, allergic rhinitis, older age, and higher baseline sIgE are clinically important risk factors for grade 2+ reactions during P-OIT. The association between pre-OIT grade 2+ initial reactions and grade 2+ reactions during P-OIT in our study is not surprising based on our clinical experience. Although there is little information to compare (given that most OIT studies have excluded patients with life-threatening reactions at baseline), our sample size is among the largest for this type of analysis and therefore provides important clues.^2^

We found a positive association between allergic rhinitis and grade 2+ reactions during P-OIT. This could be due to allergic rhinitis symptoms acting as a cofactor, lowering the threshold for a reaction and leading to a grade 2+ reaction.^5^ Further research is needed to determine whether there is a causal relationship between allergic rhinitis and increased risk of grade 2+ reactions during P-OIT.

Age at start of P-OIT was positively associated with grade 2+ reactions during P-OIT. A 12-month increase in age correlated to a 13% higher risk of having a grade 2+ reaction during P-OIT (OR = 1.13), with 95% CI ranging from a 1% decrease to a 30% increase in risk, which is clinically relevant. Similarly, older age has been shown by others to negatively affect safety during OIT; in the Chu et al^6^ meta-analysis of OIT, 11.8% of school-age patients (median age, 8.7 years) required epinephrine during P-OIT, compared to 2.7% of preschool patients in the study of Vickery et al.^7^

We identified a positive correlation between baseline sIgE and grade 2+ reactions during P-OIT, consistent with our group’s previous studies.^2^^,^^4^ The OR of 1.02 is based on a 1-unit increase in baseline IgE. Hence, a 10-unit increase in baseline sIgE correlates to a 27% higher risk of having a grade 2+ reaction during P-OIT, which is clinically relevant. Similarly, data from Yanagida et al^8^ suggest a statistically significant association between anaphylaxis during oral food challenges and higher IgE levels (OR = 2.71), which could indicate that there is a correlation between IgE levels and severity of allergic reactions.

Although we found no relationship between preexisting asthma and grade 2+ reactions during P-OIT, uncontrolled asthma is a risk factor for more severe reactions, and screening for the development of asthma is an essential part of providing OIT.^9^ It is possible that this relationship was not observed in our model because we were unable to distinguish controlled versus uncontrolled asthma, and we did not include newly diagnosed asthma after P-OIT initiation because this information was unavailable.

Our findings are limited by missing data in our data set, particularly for baseline sIgE, and we acknowledge that this is a feature of real-world data. To mitigate this limitation, we performed multiple imputations to reduce potential bias and maximize use of available information, and we also carried out 2 sensitivity analyses.

In summary, our study identified several clinically important risk factors for grade 2+ reactions during P-OIT: pre-OIT grade 2+ initial reaction, allergic rhinitis, older age, and higher baseline sIgE. These results highlight the need for individualized risk stratification for OIT—for example, consideration of slower buildup for patients with these risk factors. Future goals of this research include collection of more data to refine the model and creation of an algorithm to predict the safety of OIT for each patient on the basis of their unique profile, which could reduce the risk of moderate or severe allergic reactions and ultimately transform clinical practice.

Detailed methods are available in the Methods section in this article’s Online Repository at www.jaci-global.org.

**Clinical implication**

Retrospective analysis of 653 preschoolers receiving P-OIT identified higher grade of initial reaction, allergic rhinitis, older age, and higher baseline sIgE as risk factors for moderate/severe reactions during OIT.

## References

[1] Soller L., Abrams E.M., Carr S., Kapur S., Rex G.A., Leo S. (2021). First real-world effectiveness analysis of preschool peanut oral immunotherapy. J Allergy Clin Immunol Pract.

[2] Soller L., Abrams E.M., Carr S., Kapur S., Rex G.A., Leo S. (2019). First real-world safety analysis of preschool peanut oral immunotherapy. J Allergy Clin Immunol Pract.

[3] Patel N., Vazquez-Ortiz M., Turner P.J. (2019). Risk factors for adverse reactions during OIT. Curr Treat Options in Allergy.

[4] Soller L., Carr S., Kapur S., Rex G.A., McHenry M., Cook V.E. (2022). Real-world peanut OIT in infants may be safer than non-infant preschool OIT and equally effective. J Allergy Clin Immunol Pract.

[5] Chua G.T., Chan E.S., Soller L., Cameron S.B. (2021). Grass pollen allergy as an anaphylaxis cofactor during peanut oral immunotherapy. Ann Allergy Asthma Immunol.

[6] Chu D.K., Wood R.A., French S., Fiocchi A., Jordana M., Waserman S. (2019). Oral immunotherapy for peanut allergy (PACE): a systematic review and meta-analysis of efficacy and safety. Lancet.

[7] Vickery B.P., Berglund J.P., Burk C.M., Fine J.P., Kim E.H., Kim J.I. (2017). Early oral immunotherapy in peanut-allergic preschool children is safe and highly effective. J Allergy Clin Immunol.

[8] Yanagida N., Sato S., Takahashi K., Nagakura K.I., Asaumi T., Ogura K. (2018). Increasing specific immunoglobulin E levels correlate with the risk of anaphylaxis during an oral food challenge. Pediatr Allergy Immunol.

[9] Varshney P., Steele P.H., Vickery B.P., Bird J.A., Thyagarajan A., Scurlock A.M. (2009). Adverse reactions during peanut oral immunotherapy home dosing. J Allergy Clin Immunol.

