# Supplementary material for: Risk factors associated with safety of preschool peanut oral immunotherapy
Source: J Allergy Clin Immunol Glob. 2023 Mar 21;2(2):100094. doi: 10.1016/j.jacig.2023.100094 (PMC10510002; doi:10.1016/j.jacig.2023.100094)
Supplement: Supplementary Material [file mmc1.docx]

To address missing data, we compared the outcome (grade 2+ reactions during P-OIT) and risk factors between patients with and without missing data to explore potential bias caused by missingness. Furthermore, multiple imputation was applied to reduce the potential bias caused by missingness and maximize use of available information to preserve statistical power. All risk factors were included in the imputation model (Model AMMI). The fully conditional specification imputation algorithm was performed, where the regression method was used for all imputed continuous variables and the discriminant function method was used for all imputed classification variables. 20 imputed datasets were created. The convergence of imputation model was also assessed.

We also carried out two sensitivity analyses – 1) multivariable logistic regression model with complete case analysis (Model SA1) and 2) multivariable logistic regression model excluding risk factors with a large amount of missing data (Model SA2) to further examine different approaches to address missingness.
